# Interpretable machine learning for prediction of clinical outcomes in acute ischemic stroke

**DOI:** 10.3389/fneur.2023.1234046

**Published:** 2023-09-07

**Authors:** Joonwon Lee, Kang Min Park, Seongho Park

**Affiliations:** Department of Neurology, Haeundae Paik Hospital, Inje University College of Medicine, Busan, Republic of Korea

**Keywords:** machine learning, stroke, cerebral infarction, explainable artificial intelligence, prediction, functional outcome, modified Rankin Scale

## Abstract

**Background and aims:**

Predicting the prognosis of acute ischemic stroke (AIS) is crucial in a clinical setting for establishing suitable treatment plans. This study aimed to develop and validate a machine learning (ML) model that predicts the functional outcome of AIS patients and provides interpretable insights.

**Methods:**

We included AIS patients from a multicenter stroke registry in this prognostic study. ML-based methods were utilized to predict 3-month functional outcomes, which were categorized as either favorable [modified Rankin Scale (mRS) ≤ 2] or unfavorable (mRS ≥ 3). The SHapley Additive exPlanations (SHAP) method was employed to identify significant features and interpret their contributions to the predictions of the model.

**Results:**

The dataset comprised a derivation set of 3,687 patients and two external validation sets totaling 250 and 110 patients each. Among them, the number of unfavorable outcomes was 1,123 (30.4%) in the derivation set, and 93 (37.2%) and 32 (29.1%) in external sets A and B, respectively. Among the ML models used, the eXtreme Gradient Boosting model demonstrated the best performance. It achieved an area under the receiver operating characteristic curve (AUC-ROC) of 0.790 (95% CI: 0.775–0.806) on the internal test set and 0.791 (95% CI: 0.733–0.848) and 0.873 (95% CI: 0.798–0.948) on the two external test sets, respectively. The key features for predicting functional outcomes were the initial NIHSS, early neurologic deterioration (END), age, and white blood cell count. The END displayed noticeable interactions with several other features.

**Conclusion:**

ML algorithms demonstrated proficient prediction for the 3-month functional outcome in AIS patients. With the aid of the SHAP method, we can attain an in-depth understanding of how critical features contribute to model predictions and how changes in these features influence such predictions.

## Introductions

Physicians are concerned about the prognosis following a diagnosis of acute ischemic stroke (AIS) and must establish a long-term treatment plan (1). Traditional risk-scoring models have been used to predict clinical outcomes after AIS (2–4), but these models face challenges when learning the input of complex multi-dimensional functions. The optimal weight for one input can easily change depending on other input values (5, 6). On the other hand, machine learning (ML) algorithms have the advantage of being able to maximize information to enhance predictive accuracy while predicting complex clinical outcomes influenced by various situations and conditions.

However, the clinical application of ML models has been hindered by the interpretability challenge due to the black box problem (7, 8). In medicine, the lack of interpretability can make it difficult for clinicians to trust the predictions of the model and incorporate them into their decision-making (9). The SHapley Additive exPlanations (SHAP) is an attribution method used to provide interpretation and an intuitive understanding of the contribution of each variable to the outcome (10).

In this study, we aimed to develop and validate an ML-based model that predicts the functional outcome of patients with AIS. Furthermore, we aimed to provide interpretation and an intuitive understanding of the model using model-agnostic attribution methods.

## Materials and methods

### Study design and source of data

This study is a prognostic accuracy cohort study utilizing prospectively collected multicenter hospital-based stroke registries. The objective of this study is to develop, validate, and interpret a machine learning system for predicting 3-month functional outcomes after stroke. We utilized a derivation dataset for model training and internal validation, while external datasets were used for external validation.

The dataset from the National Information Society Agency (NIA) was used as the derivation set (https://aihub.or.kr/). This is a dataset prepared for developing an artificial intelligence model from a multicenter prospective stroke registry. It contains clinical data of patients diagnosed with AIS between January 2011 and March 2019 at the stroke centers of three university hospitals in the Republic of Korea. A diagnosis of AIS was confirmed when there was diffusion restriction on MRI. In the dataset of 6,000 patients, data for 1,717 patients for whom clinical data were unavailable were discarded. Of the 4,283 eligible patients, patients under the age of 18 years, pre-stroke modified Rankin scale (mRS) of three points or more, and who were not evaluated with follow-up mRS were excluded (11). Additional external sets were collected from a stroke center, where acute ischemic stroke patients who visited each center from September 2019 to August 2021 were screened. Ultimately, the derivation set was composed of datasets from two hospitals from the dataset of the NIA, and the remaining one dataset of the hospital along with an additional externally collected dataset became two separate external validation sets. We have selected 16 clinical features from the 31 common clinical features of the NIA dataset, based on both a feature importance algorithm and at the clinicians' discretion (see Supplementary Table S1 for further details). These selected features include initial NIHSS, END, age, BUN, WBC, prothrombin time, previous stroke, serum glucose, hemoglobins, atrial fibrillation, hypertension, diabetes mellitus, BMI, onset-to-door time, EVT, and tPA.

This study followed the TRIPOD and CLAIM reporting guidelines (12, 13). All data were anonymized using the de-identification method of the data provider (Supplementary Figure S1).

### Definition of functional outcome

A functional outcome was a binary label, defined as an unfavorable outcome if the mRS is 3 or more, and a favorable outcome if < 3 (11). The functional outcome was evaluated in-person by a neurology specialist when patients revisited the outpatient department 3 months post-ischemic stroke. For a minority of patients who could not attend the outpatient clinic, evaluations were conducted over the phone by a well-trained clinical nurse specialist.

### Data preparation

We performed outlier detection and imputation for missing values < 10% on the clinical data of the entire dataset. Two experienced clinicians, KMP and JL, checked the distributions of the values of 16 common variables and looked for levels that were not clinically feasible. Outlier candidates were replaced with NAs by clinician consensus. We then conducted multivariate imputation chained equations (MICE) on both the NA-replaced outliers and the missing values of the original data (14). After these procedures, we normalized all the data using the MinMaxScaler.

### Model development and evaluation

We implemented a supervised machine learning approach to develop a predictive model for the 3-month functional outcome of acute ischemic stroke. The modeling process engaged three tree-based machine learning algorithms: random forest, eXtreme Gradient Boosting (XGBoost), and light gradient-boosting machine (LGBM) (Supplementary Table S2). These algorithms were chosen due to their proven efficacy in calculating interaction effects among features using TreeSHAP (15).

To validate the performance of the model, we performed not only internal validation but also external validation using two separate external datasets. Initially, we randomly allocated 20% of the entire dataset as an internal test set and assigned the remaining 80% of the data to a training set. We then used a 5-fold cross-validation method to generate each model. This procedure resulted in five versions of each machine learning model, with their performance being evaluated on the internal test set. The performance of each model was determined using the mean value of the five derived probabilities (see Supplementary Figure S2). Finally, we conducted validation using the developed models on the two external datasets. The primary performance metric we employed to assess the effectiveness of the model was the area under the receiver operating characteristic curve (AUC-ROC). All analyses were performed using Python 3.10.6 and the scikit-learn 1.0.2 library.

### Interpretation of the developed models

We employed the SHAP method for interpreting the developed model. First, we calculated the SHAP values for each feature in the model that performed the best. These SHAP values were used to explain the contribution of each feature in the prediction of the model. By plotting the SHAP summary plot, we visualized the top 16 features that were most impactful in the output of the model. Second, we produced a SHAP dependency plot. This plot primarily displays two aspects. One, it shows how SHAP values change as the values of each feature change, essentially demonstrating the relationship between the features and the predictions. The other is it reveals the interactions between features. Through these two aspects, we were able to ascertain the contribution of each feature toward an unfavorable outcome and the interactions between the features.

### Statistical analysis

Values are presented as the mean ± standard deviation, median (interquartile range) for continuous variables, or as the number (%) of subjects for categorical variables, as appropriate. Comparisons of the characteristics between the two groups were performed by the chi-square test, Fisher's exact test, Wilcoxon Signed Rank test, or Cochran–Armitage trend test according to the type of the variable. To evaluate the performance of the models for discriminating 3-month functional outcome, we plotted receiver operating characteristic (ROC) curves and calculated the AUC and 95% confidence interval (CI) for each model. Differences between AUCs were compared by DeLong's test (16). Additionally, recall (sensitivity), precision (positive predictive value), accuracy, and Brier score were calculated as secondary outcome metrics. We also calculated the sensitivity and specificity value for the threshold determined by Youden's index J (J = sensitivity + specificity-1) if necessary. Statistical analyses were performed using the R package version 4.1.2. The statistical significance was defined as a two-tailed *P*-value of < 0.05.

## Results

A total of 3,687 patients were included in the derivation dataset, of which 1,123 (30.4%) had an unfavorable outcome. The external validation dataset had 360 patients: 250 in external dataset A and 110 in dataset B, with 93 (37.2%) and 32 (29.1%) unfavorable outcomes, respectively (Supplementary Figure S3). All missing values for the derivation dataset common variables were within 10% (Supplementary Figure S4). Hence, multivariate imputation-chained equations were applied to all variables (14). Baseline demographics and clinical characteristics according to the favorable and unfavorable outcomes of the dataset on which the imputation was performed are listed in Supplementary Table S3.

### Model performances

Table 1 presents the performance estimates of the ML algorithm. Among the three tree-based ML algorithms, the performance was quite similar, but XGB demonstrated the highest performance. As a result, we chose XGB as the representative ML algorithm. The area under the receiver operating characteristic curve (AUROC) values for the internal test set and external validation sets A and B of the model were 0.790 (95% confidence interval [95% CI]: 0.775–0.806), 0.791 (95% CI: 0.733–0.848), and 0.873 (95% CI: 0.798–0.948), respectively (Supplementary Figure S5). The Brier scores for the internal validation set and external validation sets A and B of the model were 0.172, 0.202, and 0.141, respectively. Model calibration was performed to assess the likelihood that a given new observation belongs to each of the known classes. The calibration slopes showed a minimal difference between the predicted and observed probability of unfavorable outcomes, indicating a good model fit (Supplementary Figure S6).

**Table 1:** Comparison of performance of the models predicting 3-month functional outcomes.

	**RF**	**LGBM**	**XGB**
**Internal test set (*****n** =* **737)**			
AUROC (95% CI)	0.789 (0.773–0.804)	0.785 (0.769–0.801)	0.790 (0.775–0.806)
Recall (95% CI)	0.695 (0.668–0.722)	0.705 (0.679–0.732)	0.756 (0.731–0.781)
Precision (95% CI)	0.553 (0.527–0.579)	0.540 (0.514–0.565)	0.525 (0.500–0.549)
Accuracy (95% CI)	0.736 (0.736–0.736)	0.727 (0.727–0.727)	0.717 (0.717–0.717)
**External validation set A (*****n** =* **250)**			
AUROC (95% CI)	0.794 (0.737–0.852)	0.787 (0.729–0.845)	0.791 (0.733–0.848)
Recall (95% CI)	0.677 (0.582–0.772)	0.806 (0.726–0.887)	0.656 (0.559–0.752)
Precision (95% CI)	0.677 (0.582–0.772)	0.551 (0.468–0.635)	0.663 (0.566–0.760)
Accuracy (95% CI)	0.760 (0.759–0.761)	0.684 (0.682–0.686)	0.748 (0.747–0.749)
**External validation set B (*****n** =* **110)**			
AUROC (95% CI)	0.860 (0.788–0.940)	0.869 (0.795–0.943)	0.873 (0.798–0.948)
Recall (95% CI)	0.875 (0.760–0.990)	0.844 (0.718–0.970)	0.875 (0.760–0.990)
Precision (95% CI)	0.609 (0.468–0.750)	0.643 (0.498–0.788)	0.667 (0.524–0.809)
Accuracy (95% CI)	0.800 (0.797–0.803)	0.818 (0.816–0.821)	0.836 (0.834–0.839)

AUROC, area under the receiver operating characteristic curve; RF, random forest; LGBM, light gradient boosting model; XGB, eXtreme Gradient Boosting.

### Identification of important features

**Figure 1:**
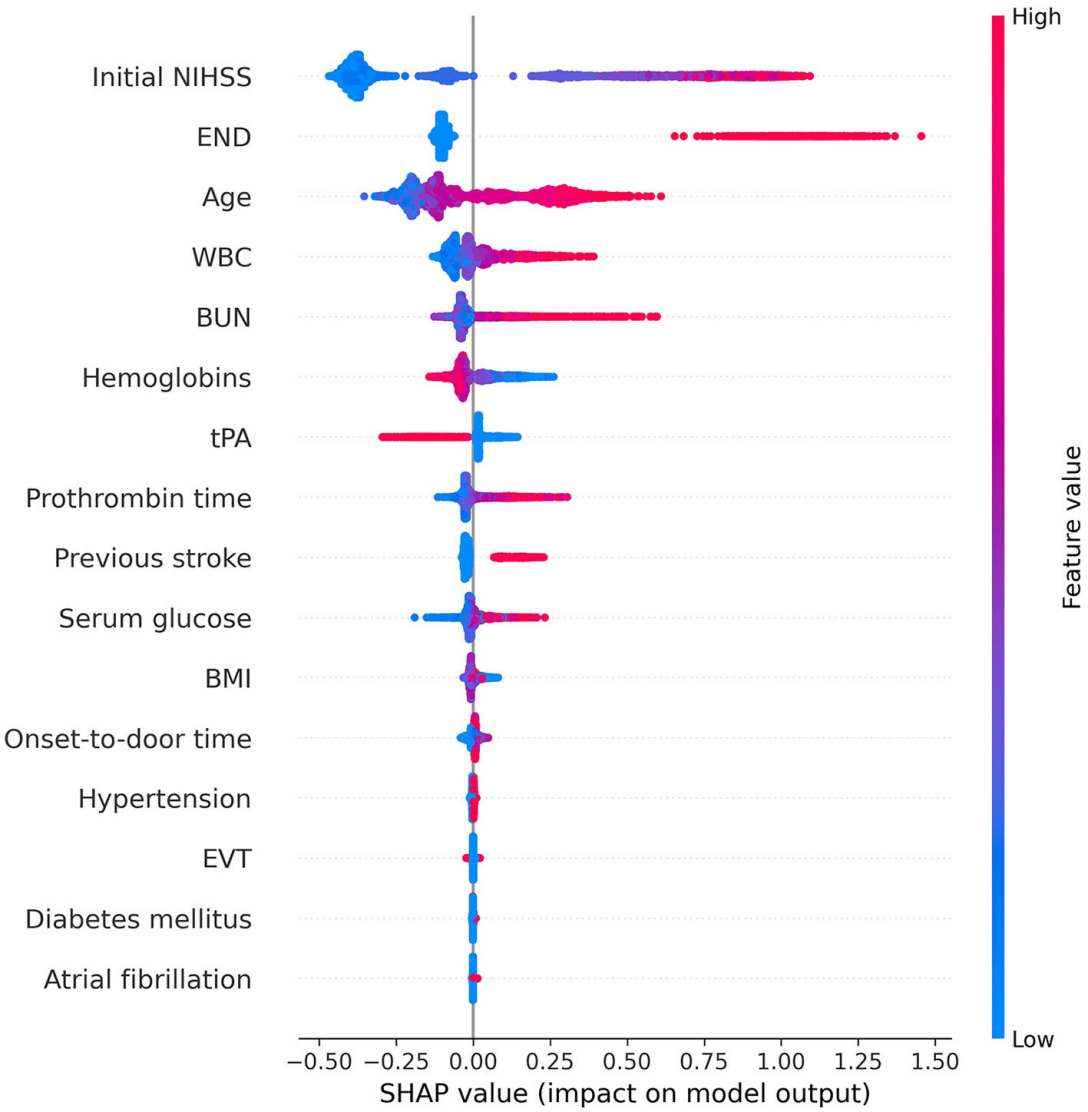
Feature importance of the gradient boosting model using SHAP. In this summary plot, the redder the value, the larger the value of the feature, and the bluer the value, the smaller the value. In addition, if the SHAP value increases in the positive direction based on 0, the probability of predicting positive increases, and if the SHAP value increases in the negative direction, the probability of predicting negative increases. The higher the initial NIHSS score, the higher the probability of predicting positive for unfavorable outcomes. However, when the hemoglobin level is high or tPA is used, the probability of predicting a negative outcome increases. We obtained information on the importance and contribution of each patient through SHAP. This helps in understanding how much each feature contributes to the model prediction and how that contribution changes. This information enhances the interpretability of the model and can provide insightful guidance for feature selection or model tuning. NIHSS, National Institutes of Health Stroke Scale; END, early neurologic deterioration; WBC, white blood cell; BUN, blood urea nitrogen; tPA, tissue plasminogen activator; BMI, body mass index; EVT, endovascular treatment; SHAP, SHapley Additive exPlanations.

The SHAP summary plot illustrated the influence of each feature on an unfavorable outcome (Figure 1). In the descending order, the risk factors contributing most significantly to the prediction of an unfavorable outcome were initial NIHSS, early neurologic deterioration, age, WBC, BUN, hemoglobins, PT, use of tPA, and previous stroke. The initial NIHSS, early neurologic deterioration, and age provided notably larger contributions to the unfavorable outcome prediction compared to the other risk factors. The variables hemoglobins and the use of tPA showed a negative correlation with the incidence of an unfavorable outcome.

**Figure 2:**
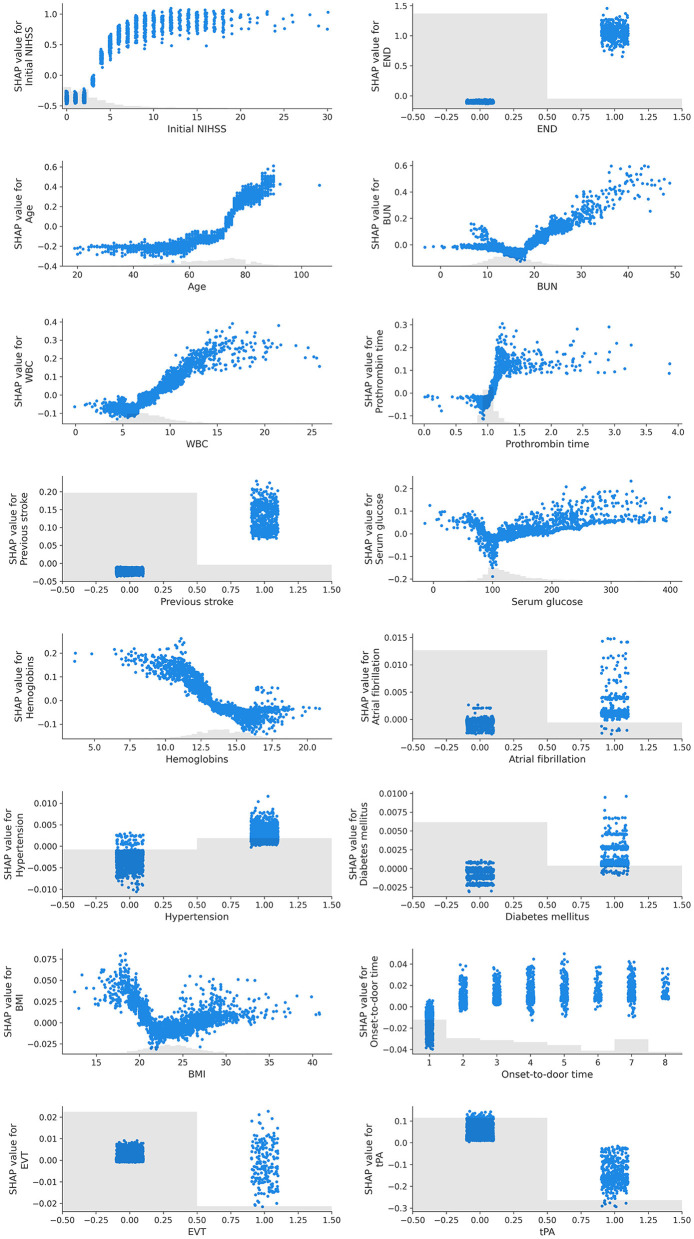
SHAP dependency plots of features. In this graph, the x-axis represents the values of a specific feature, while the y-axis represents the SHAP values of that feature. Each point signifies an individual data point, and its position denotes the value of the feature and the impact that this value has on the prediction. For instance, if the SHAP value consistently increases as the feature value rises, it can be interpreted that the feature has a positive influence on the prediction of the model. Conversely, if the SHAP value decreases as the feature value increases, the feature can be understood to negatively affect the prediction. Furthermore, the SHAP dependency plot is useful for visualizing non-linear relationships. For example, if the plot exhibits a U or S shape, it indicates a non-linear relationship between the feature and the prediction.

The SHAP dependency plot showed how the SHAP values change as each feature value changes (Figure 2). Initial NIHSS, END, age, BUN, WBC, PT, previous stroke, hypertension, and diabetes mellitus indicated a positive correlation with the unfavorable outcome when the feature values were positive or increasing. On the contrary, hemoglobins, atrial fibrillation, and the use of tPA exhibited an opposite influence.

We identified certain features exhibiting non-linear relationships with the outcome. Age had a progressively greater positive influence as it increased, whereas the initial NIHSS score followed a sigmoid curve with a positive correlation. BUN, serum glucose, and BMI showed a U-shaped or V-shaped curve, indicating an increase in the contribution to an unfavorable outcome when the values deviated from what is known to be a normal range.

### Identification of interaction between features

**Figure 3:**
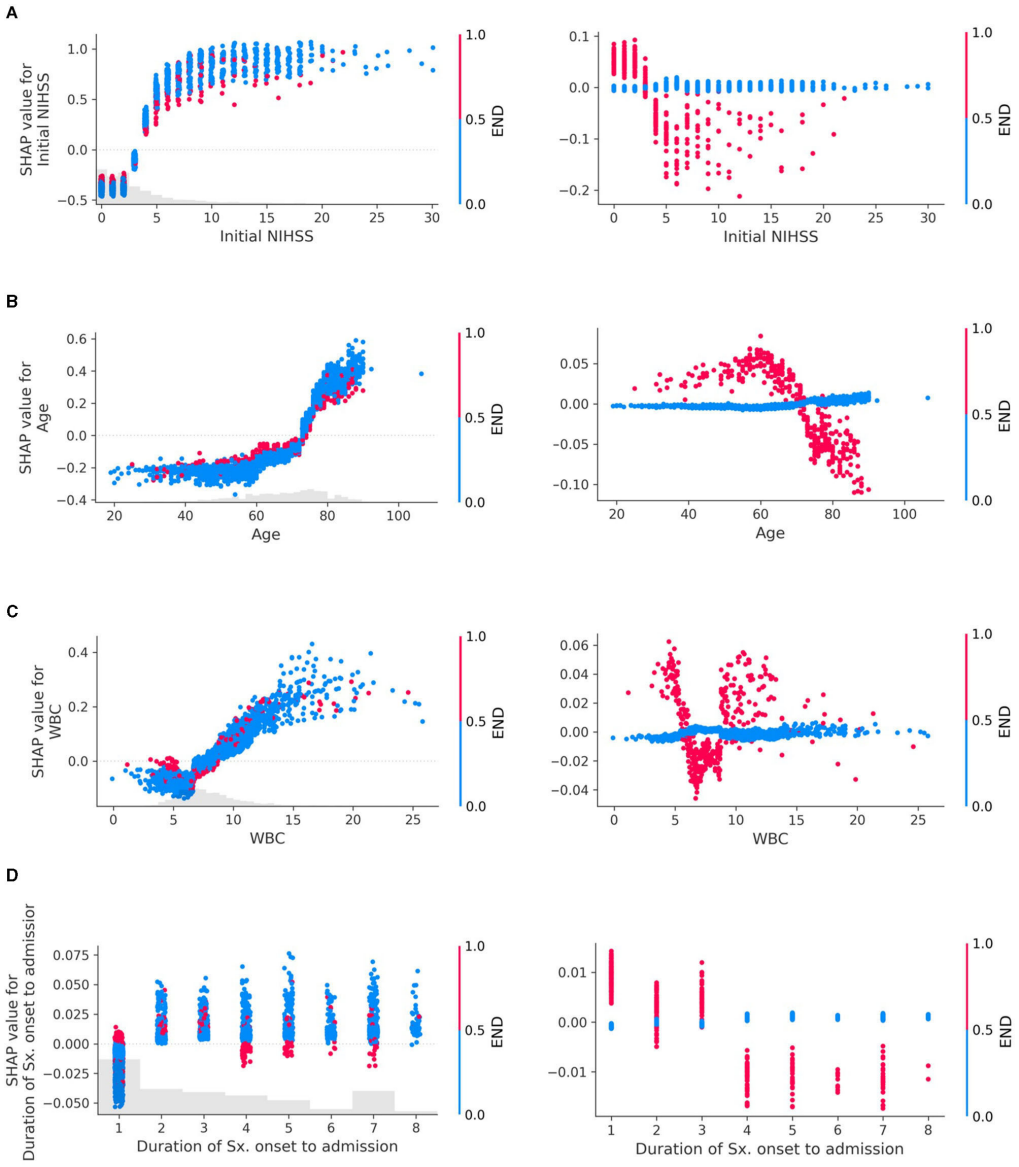
Interaction plots of early neurologic deterioration (END) with **(A)** Initial NIHSS, **(B)** Age, **(C)** White Blood Cell (WBC) count, and **(D)** Duration of symptom onset to admission. Interactions in SHAP dependency plots visually represent how the SHAP values of one feature change as the values of another feature change. The left column of the plot depicts both the main effects and the interaction effects, while the right column isolates and displays only the interaction effects. In the left column, each dot represents an individual data point, with its position indicating the value of a specific feature and the corresponding SHAP value reflecting its impact on the prediction. These dots are colored according to the values of another feature, illustrating how the relationship between a particular feature and the prediction depends on the values of another feature. If these patterns change depending on the color of the dots (i.e., the values of the other feature), it indicates an interaction between the two features. For example, if dots of a certain color tend to show an increase in SHAP values as the feature value increases, but dots of other colors show a stronger, weaker, or even opposite trend, and this suggests an interaction between the two features. The plot in the right column, drawn using only interaction values, emphasizes the interaction effects more clearly, which may not have been as evident in the left column plot. The larger the absolute value of the interaction (indicated on the y-axis), the stronger the interaction between the two features, indicating that these two features are more closely related to the prediction of the model.

The SHAP dependency plot can depict the effect of a specific feature on the prediction by using different colors to represent the values of other features. By observing these color patterns, we can explore the interactions between two features that influence the outcome (Figure 3).

END exhibited clear interactions with several features. When the initial NIHSS was < 3, the absence of END had a negative association with the unfavorable outcome compared to its presence. However, for NIHSS scores of 3 or higher, the absence of END contributed more to an unfavorable outcome than its presence (Figure 3A). In patients aged 70 years or above, the occurrence of END contributed less to an unfavorable outcome compared to those under 70 years (Figure 3B). When WBC was outside the normal range (6,000–9,000/mL), the presence of END showed a positive interaction with the unfavorable outcome (Figure 3C). Patients who arrived within 12 h from symptom onset exhibited interactions with END (Figure 3D). Additional examples of interaction effects between features, other than END, can be found in the Supplementary Figure S7.

### Individual patient outcome interpretations

SHAP allows for not only global interpretations of a feature's contribution to the outcome across the entire dataset but also local interpretations for individual patients. As illustrated in Supplementary Figure S8, the patient was predicted to not have an unfavorable 3-month functional outcome. The history of a previous stroke, an age of 74 years, and a WBC count of 8,600/mL all contributed to an unfavorable outcome. However, a lower NIHSS score of 1, the absence of END, and a hemoglobin level of 13.9 had greater contributions to a more favorable outcome. These local interpretations allow us to comprehend why the model predicted a specific outcome for this particular patient.

## Discussion

In this study, we demonstrated the application of machine learning (ML) algorithms to predict the functional outcomes following an acute ischemic stroke. Our model provided an explanation not only based on individual patient data but also utilized the entire dataset to visualize the contribution and directionality of specific input features through SHAP.

The features that contributed most significantly to our study were initial NIHSS, END, and age, which are well-known risk factors for stroke functional outcomes and have been consistently reported in previous studies (2, 17–20). In the case of a strong linear relationship between two variables where the outcome variable is binary, the predicted probabilities can manifest as a sigmoid pattern (21, 22). Interestingly, in our study, initial NIHSS showed a sigmoid curve relative to the 3-month binary functional outcome (Figure 2).

In the prediction of unfavorable outcomes, END exhibited clear interactions with most variables. When the initial NIHSS was < 3 points, the absence of END indicated a negative association with unfavorable outcomes. However, in cases where NIHSS was 3 or higher, the absence of END contributed more to unfavorable outcomes. While this finding may seem counterintuitive, it can be understood when considering that the most common cause of END is a progressive stroke, such as lacunar infarction (23). The mechanism of this progression may well be related to the concept of branch atheromatous disease, suggesting that a hypoperfused area in the perforating arteriole region may deteriorate after admission, leading to the occurrence of END, particularly in patients who were admitted with lower NIHSS scores.

This notion aligns with the results from a previous cohort study conducted on patients with lacunar infarction, where END was associated with worse functional results at 90 days, and low NIHSS scores at admission along with low perfusion lesions predicted END (18). Our findings corroborate these results. Furthermore, the same cohort study suggested that patients who received IVT treatment demonstrated improved functional outcomes at 90 days, and a conclusion is also reflected in our study.

As shown in Figure 3D, the presence of END within 12 h of admission demonstrated a positive interaction with unfavorable outcomes, whereas it exhibited a negative interaction in patients who arrived later. This discrepancy could be attributed to the fact that patients arriving at the hospital quickly are more likely to have END identified. However, for those arriving later, even if END has occurred, it may not be recorded as such in the data if the event happened outside the hospital, thus potentially explaining this observed interaction.

The other features, while not as widely recognized as risk factors for 3-month functional outcomes as the ones previously mentioned, showed consistency in our model interpretation with existing research results (Figure 2). The *post-hoc* analysis of the Enhanced Control of Hypertension and Thrombolysis Stroke Study (ENCHANTED), which indicated that hyperglycemia and increased WBC count independently relate to poor functional outcomes (24), was reproduced in our study. Interestingly, lower WBC counts at admission were associated with better functional outcomes regardless of treatment (25), and the previously observed association of increased WBC with poor functional outcomes seemed to be reproduced in our results as a U-shaped impact pattern.

Consistent with previous studies suggesting a potential link between increased BUN and unfavorable outcomes (26), our study also found an association between increased BUN and unfavorable outcomes. BUN exhibited a V-shaped pattern, which seems to be a plausible non-linear result when combined with previous findings that a decrease in BUN is associated with better neurological improvement (27).

Our study found that a decrease in hemoglobin contributed significantly to unfavorable outcomes. Previous studies have also found significant associations between abnormal hemoglobin levels and poor outcomes (28, 29).

The duration of symptoms to hospital admission within 3 h tended to decrease the likelihood of unfavorable outcomes but showed a positive association thereafter. However, this influence slightly decreased as the duration increased. This likely reflects the impact of being able to use rtPA for those who arrived within 3 h.

In Figure 1, EVT showed relatively low importance for the model, but the dependency plot revealed its association with a favorable outcome in patients with an initial NIHSS value of 6 or more (Supplementary Figure S7c). This aligns with guidelines suggesting that performing EVT on patients with an initial NIHSS score of 6 or higher correlates with better prognoses (30). However, it is necessary to interpret with caution, considering that features with low contributions might have limited clinical significance for the model.

We looked at the interpretation between the classical generalized linear models and the interpretation of ML-based SHAP. Referring to the estimates from the multivariable logistic regression model based on the dataset used in this study, there was no considerable difference in the contributions of major features derived from the SHAP of the XGB model (Figure 1, Supplementary Figure S9). The overall consistency in the results from both approaches in our dataset can be attributed to the following reasons. As shown in Figure 2, variables considered crucial in this study (initial NIHSS, Age, and END) did not exhibit complex non-linear or multi-dimensional relationships. Datasets with such simple inter-variable relationships are likely to result in no substantial difference in predictive performance between traditional and ML models. Indeed, the AUC of the logistic regression model was not notably different from the XGB model. The AUC for the logistic regression was 0.792 (0.776–0.807) for the internal test set, 0.795 (0.737–0.853) for external validation set A, and 0.893 (0.829–0.956) for set B. These AUC values from the logistic regression did not show a statistically significant difference when compared to the AUC of the XGB model (*p* = 0.793, 0.738, and 0.247 for the internal test set, external validation sets A and B, respectively). However, in datasets with more complex relationships and interactions between variables, the interpretation results and performance of these two models might differ. Ultimately, understanding the characteristics and structure of the data and choosing the appropriate model may be pivotal in clinical research and practical decision-making.

Nevertheless, interpretable methods such as SHAP provides added value. Through the use of SHAP, we can visually understand not only the intensity of the contribution of risk factors to outcomes but also the non-linear contribution of specific risk factors and the interactions between variables. These are insights that were not discernible in previous traditional statistical methods. Through such analysis, we gain a deep understanding of how key features contribute to model predictions and how changes in their values impact those predictions. For example, we can not only reproduce the results of previous studies but also decipher hidden implications within those results. Furthermore, we can identify lesser-known risk factors. In this way, interpretable methods can provide insights about characteristics observed in clinical settings, potentially improving model performance and facilitating its use in medical practice.

Our research has several distinct advantages over previous studies. Earlier research efforts particularly showed limitations in their generalizability. This was mainly due to the limited amount of data relative to the number of features and the absence of external validation (31–33). However, our study overcame these limitations by utilizing a much broader sample and conducting both internal cross-validation and external validations.

Furthermore, while past research lacked clear methodologies or approaches to the interpretability of ML models, our research sought to address this gap by employing SHAP. Through this, we were able to delve deeper into the interactions and relationships among the variables used in our study. We believe that our research will significantly contribute to expanding the current knowledge on post-stroke management in clinical decision support systems powered by AI.

Our study has several limitations. First, due to the multicenter retrospective nature of the investigation, the performance results may be inadequate for determining the robustness of the ML model for clinical utility. Well-designed prospective cohort studies are required to provide clear evidence for clinical use.

Second, the performance of our model in some external datasets was superior to that in internal validation. There could be multiple explanations for this phenomenon: (1) If the training data contain many noises or outliers, the model may overfit by learning these noises. In this case, if the test data are cleaner or better reflect the general pattern, the performance on these datasets may be better; (2) The performance results could also vary depending on the evaluation metric used. Particularly, if the distribution of training data and test data differ, the same model can have varying performance depending on the dataset. In such a case, the test dataset could receive a higher score in a particular evaluation metric; and (3) If the model is robust, that is, if it can capture specific patterns or trends not present in the training data, it can enhance performance in the test data. This happens because the model can respond well to new patterns that were not observed in the training data. If the model is not overfit and is appropriately generalized, it can have a high predictive capability for new data.

Third, our study primarily employed a cross-sectional research design, focusing on predicting short-term outcomes. While this offers immediate insights, it captures data at a single point in time, potentially limiting our ability to track changes over time. Additionally, this approach might not provide a comprehensive perspective on how these outcomes might evolve or persist in the long run. A longitudinal study on the long-term outcomes of stroke should be undertaken as it could shed light on the temporal patterns and variations, granting deeper insights into the cause-and-effect relationships.

Fourth, while SHAP helps infer the importance of features for a given model, there may be inherent problems in the SHAP inferences if the model is poorly developed or trained. However, we developed our model using a large dataset and also carried out external validation.

In conclusion, machine learning algorithms, specifically the tree-based model, can be used to predict the 3-month functional outcome in patients with acute ischemic stroke. Through the utilization of the SHAP method, we can gain an in-depth understanding of how critical features contribute to model predictions and how their changes influence these predictions. Future work should focus on refining the model, exploring additional predictive features, and validating these findings in prospective cohort studies.

## Data availability statement

The data analyzed in this study is subject to the following licenses/restrictions: access to the data from the National Information Society Agency (https://aihub.or.kr/) has been granted, but the data is only available for review by domestic residents who have received approval. Requests to access these datasets should be directed to SP, risepsh@gmail.com.

## Ethics statement

The studies involving humans were approved by Institutional Review Board (IRB No. 2021-09-025-006) of Haeundae Paik Hospital. The studies were conducted in accordance with the local legislation and institutional requirements. The Ethics Committee/institutional review board waived the requirement of written informed consent for participation from the participants or the participants' legal guardians/next of kin because the design of the study was retrospective.

## Author contributions

JL: data curation and manuscript editing. KMP: data curation. SP: study design and concept, data curation and analysis, and manuscript editing. All authors contributed to this article and approved the final manuscript.
