# Supplementary material for: Interpretable machine learning for prediction of clinical outcomes in acute ischemic stroke
Source: Front Neurol. 2023 Sep 7;14:1234046. doi: 10.3389/fneur.2023.1234046 (PMC10513028; doi:10.3389/fneur.2023.1234046)
Supplement: Supplementary file 1 [file Data_Sheet_1.DOCX]

Supplemental Materials

Interpretable machine learning for prediction of clinical outcome in acute ischemic stroke

Joonwon Lee^1^, Kang Min Park^1^, Seongho Park^1^

*1 Department of Neurology, Haeundae Paik Hospital, Inje University College of Medicine, Busan, Republic of Korea*

**Correspondence:**

Seongho Park, MD

Department of Neurology, Haeundae Paik Hospital, Inje University College of Medicine, Haeundae-ro 875, Haeundae-gu, Busan, Republic of Korea

Postcode: 48108

Tel.: +82-51-797-2082

ORCID : 0000-0002-0504-2539

## Table S1. Common clinical variables

| **Demographics** | Smoking |
| --- | --- |
| Age | Atrial fibrillation |
| Gender | History of stroke |
| **Baseline variables** | History of cancer |
| BMI – kg/m^2^ | History of coronary heart disease |
| Systolic BP – mmHg | History of peripheral artery disease |
| Diastolic BP – mmHg | **Laboratory examinations**^‡^ |
| **Stroke assessment and treatment** | WBC – 10^9^/L |
| Pre-stroke mRS | Hemoglobin – g/dL |
| NIHSS score at baseline | Hematocrit – ratio |
| TOAST | PT – INR |
| END* | Serum glucose^¶^ – mg/dL |
| Recanalization therapy^†^ | BUN – mg/dL |
| **Stroke risk factors** | Creatinine – mg/dL |
| Stroke onset to admission delay | HDL – mg/dL |
| Hypertension | LDL – mg/dL |
| Diabetes mellitus | Total cholesterol – mg/dL |
| Dyslipidemia | Triglycerides – mg/dL |

BMI, body mass index; mRS, modified Rankin Scale; NIHSS, National Institutes of Health Stroke Scale; TOAST, Trial of Org 10172 in Acute Stroke Treatment^1^; END, early neurologic deterioration.

* END was defined as an increase in the NIHSS score of 1 or more from admission for acute cerebral infarction until discharge.

† Whether acute recanalization treatment, such as intravenous tissue plasminogen activator or intra-arterial thrombectomy, was performed.

‡ All laboratory examinations were based on the results of blood samples taken at the time of admission.

¶ Random serum glucose regardless of fasting status

## Figure S1. De-identification method.


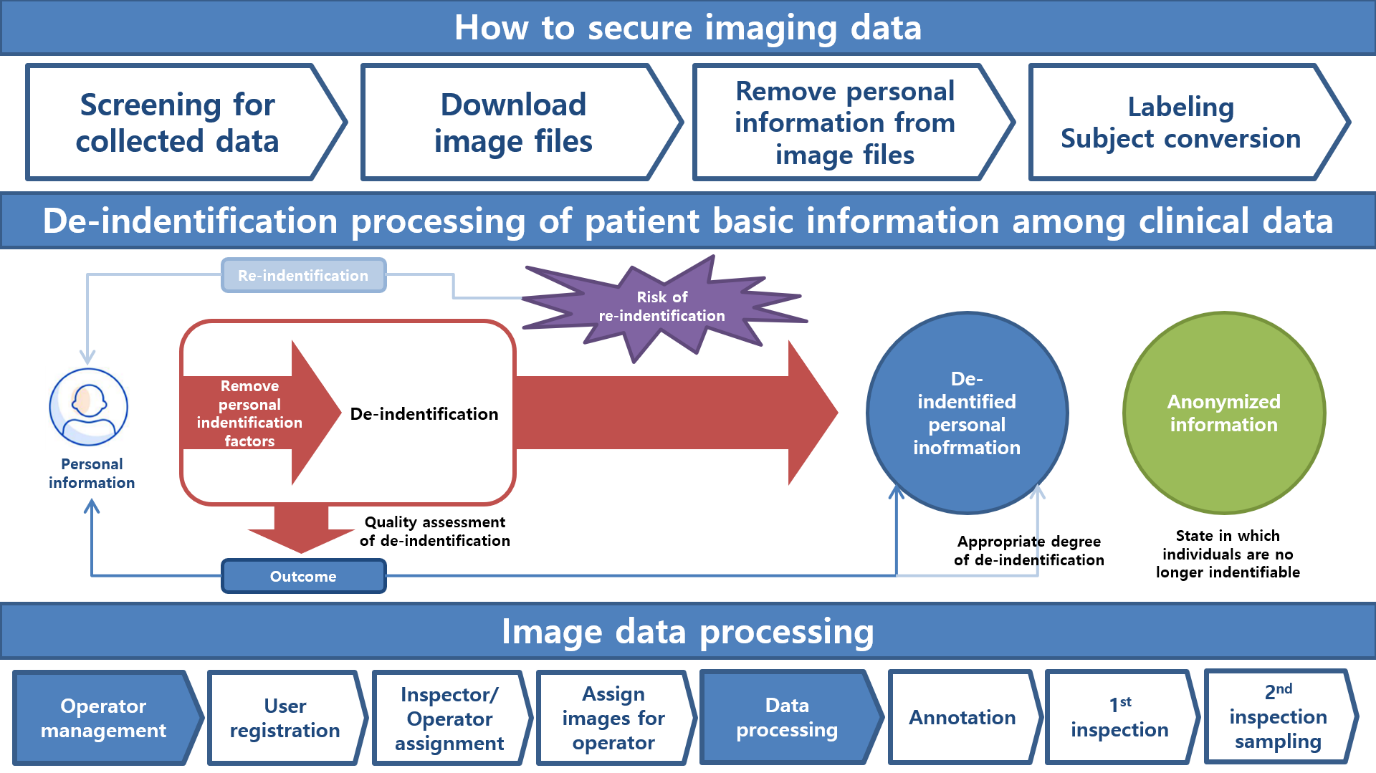


## Table S2. Tuned hyperparameter for the machine learning models.

| Algorithm | Parameters |
| --- | --- |
|  | |
| RF | n_estimators: 120, max_depth: 4, n_jobs: -1 |
| XGB | n_estimators: 200, eta (also known as learning_rate): 1e-2, max_depth: 4, subsample: 0.7, scale_pos_weight: 0.5, nthread: -1, gamma: 0.3, colsample_bytree: 0.8 |
| LGBM | n_estimators: 200, learning_rate: 1e-2, max_depth: 4, num_leaves: 5, subsample: 0.7 |

RF, random forest; XGB, extreme gradient boosting; LGBM, light gradient boosting model

## Figure S2. K-fold cross validation.


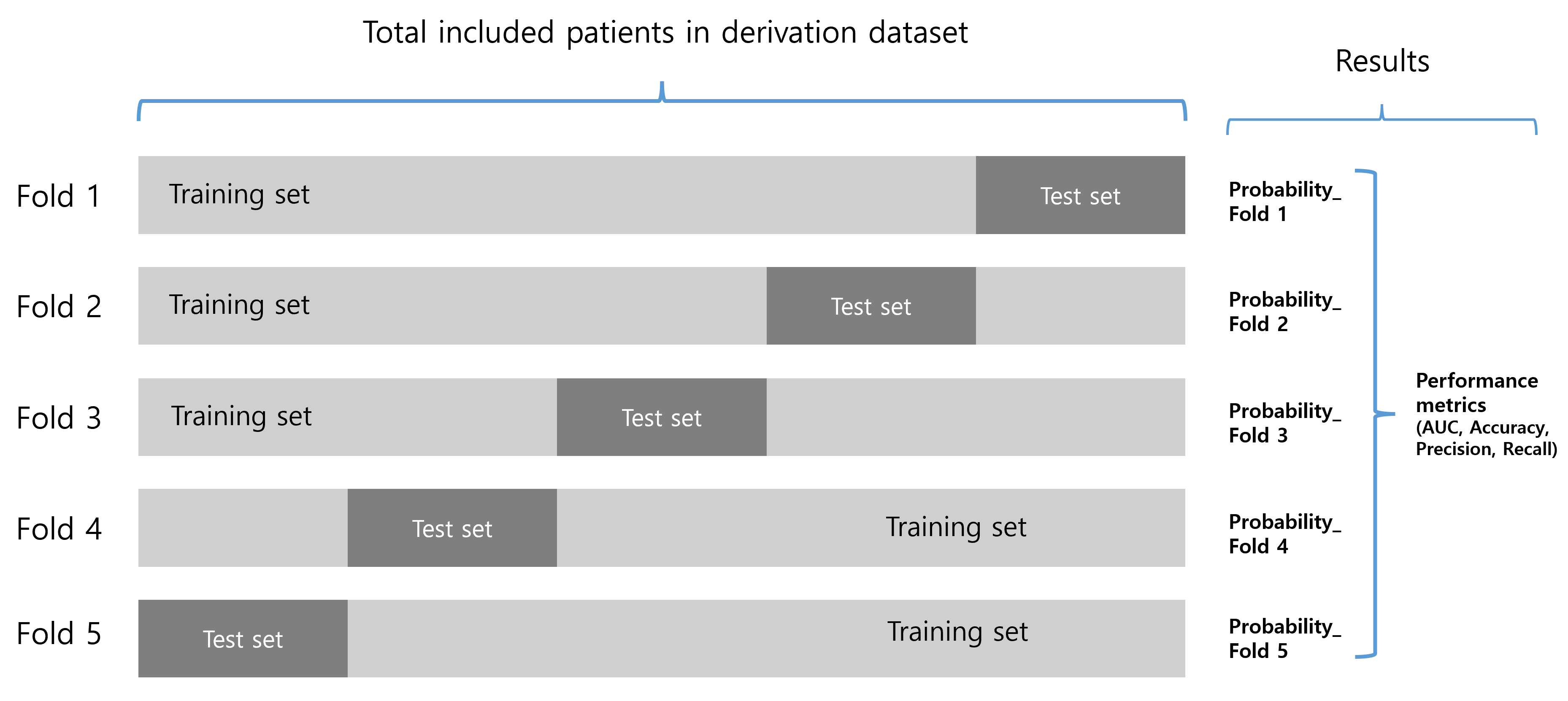


# Results

## Figure S3. Patient flowchart of derivation and external datasets.

1. Patient flowchart of the derivation dataset.


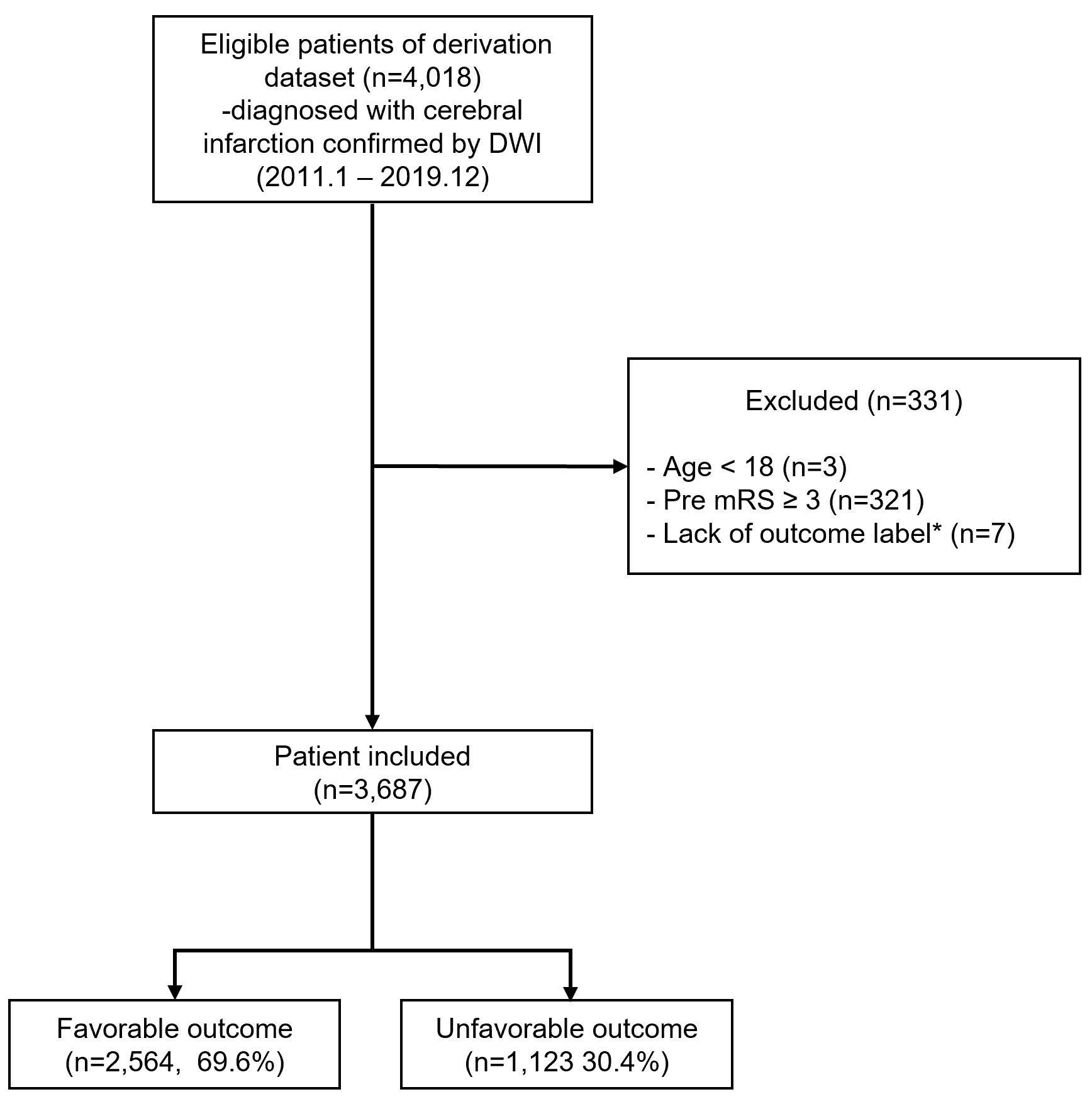


1. Patient flowchart of the external validation dataset (hospital A).


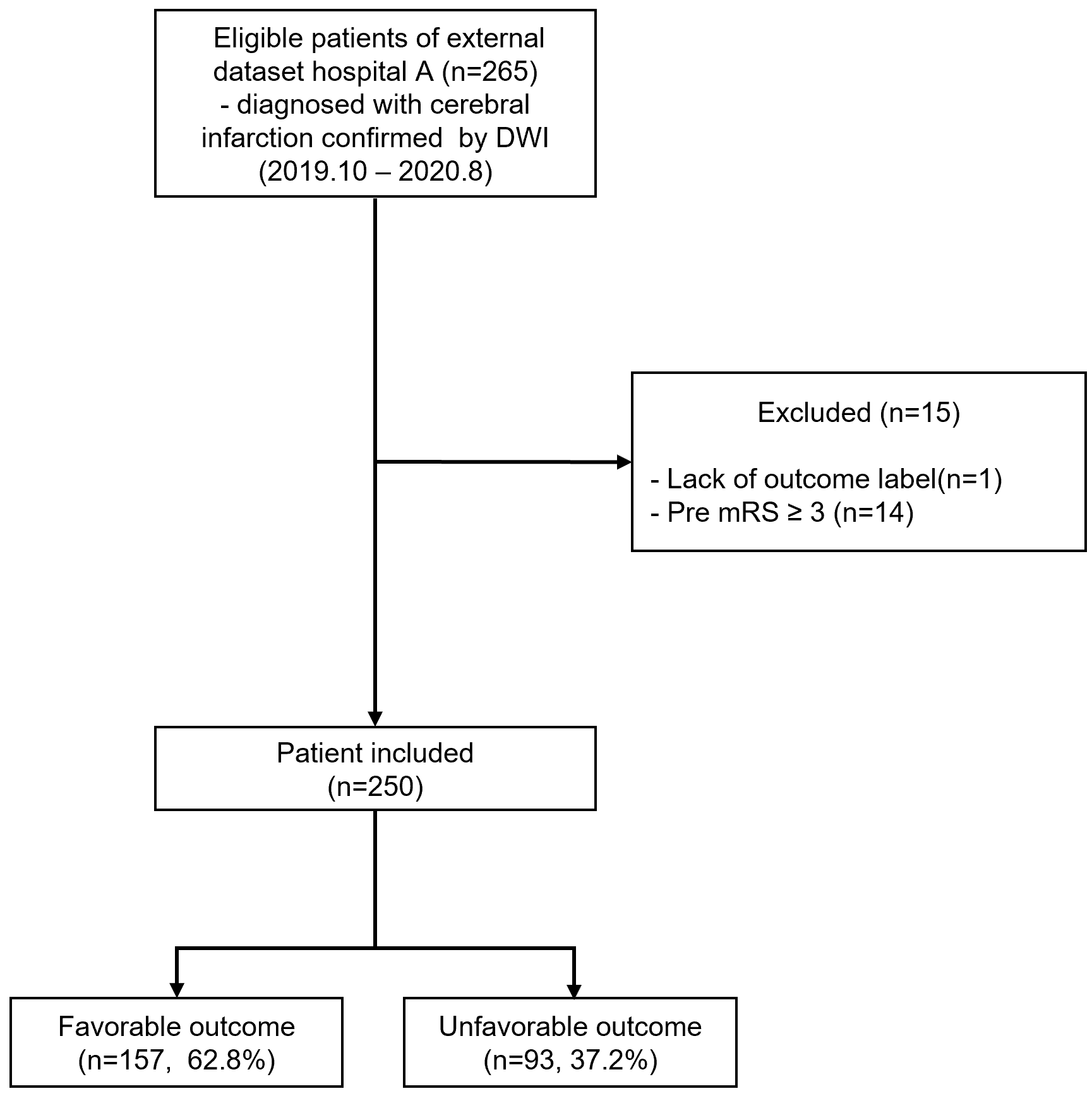


1. Patient flowchart of the external validation dataset (hospital B).


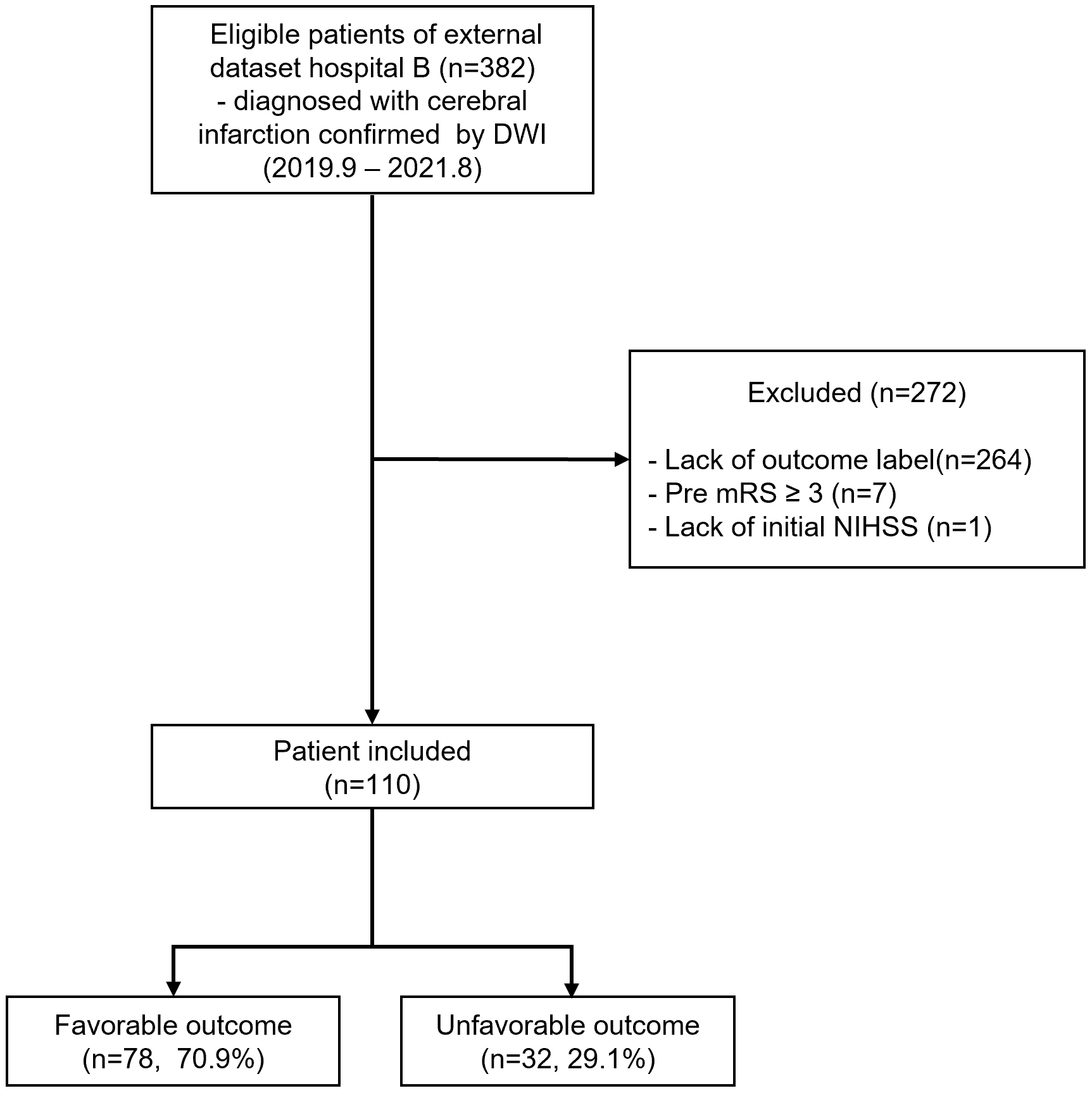


* The outcome label indicates a 3-month modified Rankin score.

n, number of patients; DWI, diffusion-weighted imaging; Pre-mRS, pre-stroke modified Rankin score; favorable outcome, month modified Rankin score ≤ 2; unfavorable outcome, 3-month modified Rankin score > 2.

## Figure S4. Missing value patterns according to the clinical variables.


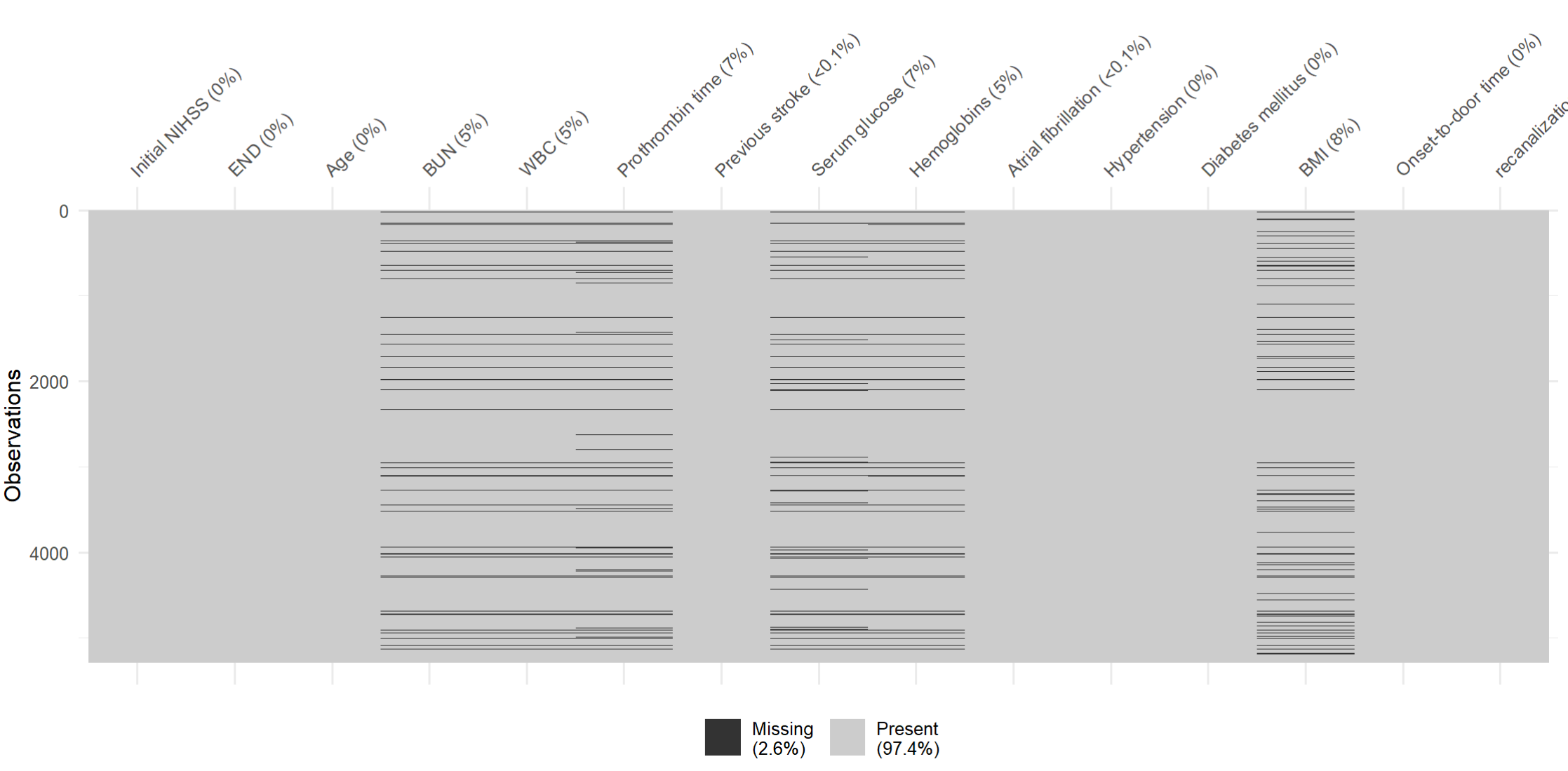


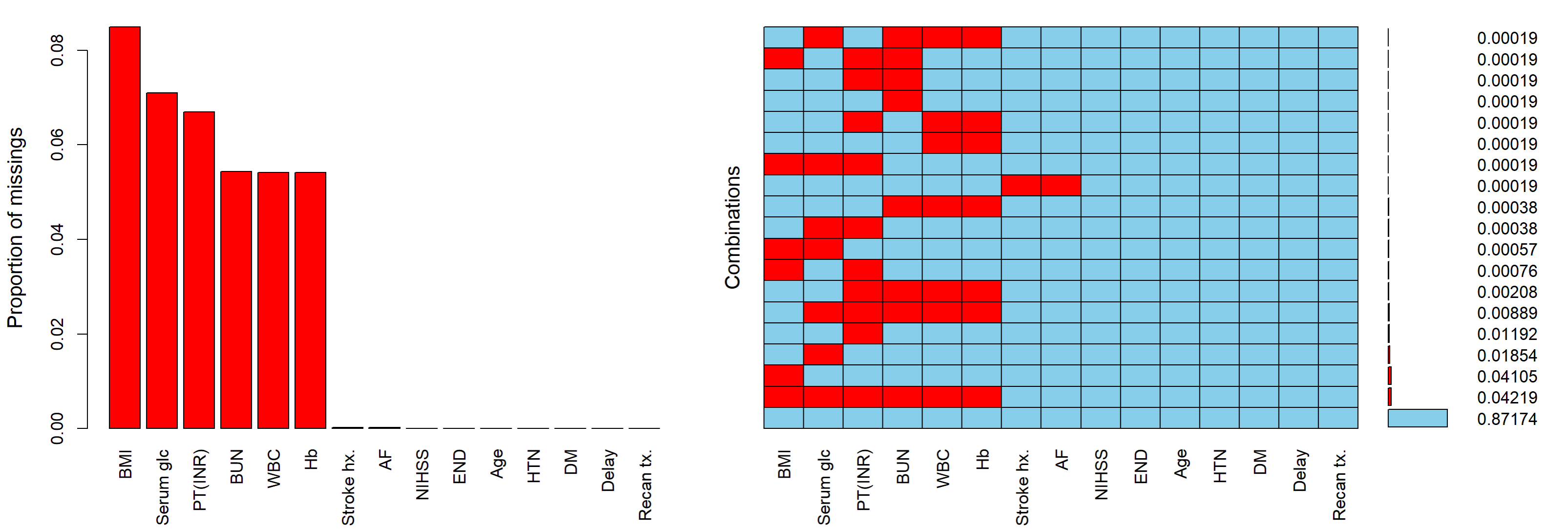


## Table S3. Baseline characteristics of the (a) derivation dataset, (b) external dataset A and (c) external dataset B after imputation.

### Derivation dataset

|  | **Favorable outcome** | **Unfavorable outcome** |  |
| --- | --- | --- | --- |
| **Variables** | (N = 2564) | (N = 1123) | P-value |
| Male – no. (%) | 1587 (61.9) | 607 (54.1) | <0.001 |
| Age – year | 65.6 ± 12.5 | 71.6 ± 11.3 | <0.001 |
| BMI – kg/m^2^ | 24.0 ± 3.2 | 23.5 ± 3.3 | <0.001 |
| Pre-stroke mRS |  |  | 0.006 |
| 0 | 1950 (76.1) | 824 (73.4) |  |
| 1 | 431 (16.8) | 184 (16.4) |  |
| 2 | 183 (7.1) | 115 (10.2) |  |
| NIHSS score  at baseline (IQR) | 2 (1-3) | 4 (2-8) | <0.001^a^ |
| TOAST – no. (%) |  |  |  |
| LAA | 1156 (32.7) | 615 (35.3) |  |
| SVO | 735 (20.8) | 275 (15.8) |  |
| CE | 534 (15.1) | 355 (20.4) |  |
| Other determined | 76 (2.1) | 35 (2.0) |  |
| Undetermined | 1034 (29.2) | 460 (42.8) |  |
| Onset to admission delay – no. (%) |  |  | 0.056^b^ |
| < 3 h | 842 (32.8) | 362 (32.2) |  |
| 3-6 h | 361 (14.1) | 196 (17.5) |  |
| 6-12 h | 336 (13.1) | 152 (13.5) |  |
| 12-24 h | 287 (11.2) | 133 (11.8) |  |
| 24-36 h | 224 (8.7) | 89 (7.9) |  |
| 36-48 h | 83 (3.2) | 33 (2.9) |  |
| 48 h-1 week | 378 (14.7) | 136 (12.1) |  |
| >1week | 53 (2.1) | 22 (2.0) |  |
| END – no. (%) | 113 (4.4) | 238 (21.2) | <0.001 |
| Acute treatment – no. (%) |  |  | <0.001 |
| No treatment | 2272 (88.6) | 929 (82.7) |  |
| IV tPA | 186 (7.3) | 112 (10.0) |  |
| IA Thrombectomy | 40 (1.6) | 46 (4.1) |  |
| Both | 66 (2.6) | 36 (3.2) |  |
| Hypertension – no. (%) | 1133 (44.2) | 594 (52.9) | <0.001 |
| Diabetes mellitus – no. (%) | 562 (21.9) | 290 (25.8) | 0.01 |
| Dyslipidemia – no. (%) | 165 (6.4) | 81 (7.2) | 0.384 |
| Atrial fibrillation – no. (%) | 359 (14.0) | 219 (19.5) | <0.001 |
| Smoking – no. (%) |  |  | <0.001 |
| Never | 1743 (68.0) | 870 (77.5) |  |
| Current | 524 (20.4) | 150 (13.4) |  |
| Ex-smoker (>5yr) | 108 (4.2) | 50 (4.5) |  |
| Ex-smoker (<5yr) | 189 (7.4) | 53 (4.7) |  |
| Previous history – no. (%) |  |  |  |
| Stroke – no. (%) | 332 (12.9) | 245 (21.8) | <0.001 |
| Cancer | 6 (0.2) | 9 (0.8) | 0.021^c^ |
| Coronary heart disease | 153 (6.0) | 79 (7.0) | 0.219 |
| Peripheral artery disease | 6 (0.2) | 7 (0.6) | 0.076^c^ |
| WBC – 10^9^/L | 7.9 ± 2.7 | 8.4 ± 3.1 | <0.001 |
| Hemoglobin – g/dL | 14.0 ± 1.8 | 13.4 ± 1.9 | <0.001 |
| Hematocrit – ratio | 40.9 ± 5.1 | 39.5 ± 5.7 | <0.001 |
| PT – INR | 1.0 ± 0.2 | 1.1 ± 0.2 | <0.001 |
| Serum glucose – mg/dL | 136.0 ± 53.3 | 139.9 ± 57.3 | 0.045 |
| BUN – mg/dL | 15.7 ± 5.7 | 17.4 ± 7.3 | <0.001 |
| Creatinine – mg/dL | 0.9 ± 0.5 | 0.9 ± 0.5 | 0.003 |
| HDL – mg/dL | 45.1 ± 16.1 | 44.5 ± 12.4 | 0.240 |
| LDL – mg/dL | 111.7 ± 38.5 | 109.6 ± 40.8 | 0.140 |
| Total cholesterol – mg/dL | 176.0 ± 43.8 | 172.5 ± 46.7 | 0.03 |
| Triglycerides – mg/dL | 127.4 ± 79.6 | 115.2 ± 75.6 | <0.001 |
| Systolic BP – mmHg | 141.8 ± 25.9 | 143.7 ± 26.5 | 0.042 |
| Diastolic BP – mmHg | 84.8 ± 14.2 | 84.3 ± 14.8 | 0.321 |

### b) External dataset A

|  | **Favorable outcome** | **Unfavorable outcome** |  |
| --- | --- | --- | --- |
| **Variables** | (N = 157) | (N = 93) | P-value |
| Age – year | 64.5 ± 12.4 | 73.5 ± 11.9 | <0.001 |
| BMI – kg/m^2^ | 24.6 ± 3.3 | 23.2 ± 3.2 | 0.001 |
| NIHSS score  at baseline (IQR) | 1 (0-3) | 3 (1-6) | <0.001^a^ |
| Onset to admission delay – no. (%) |  |  | 0.933^b^ |
| < 3 h | 35 (22.3) | 26 (28.0) |  |
| 3-6 h | 33 (21.0) | 12 (12.9) |  |
| 6-12 h | 25 (15.9) | 14 (15.1) |  |
| 12-24 h | 16 (10.2) | 14 (15.1) |  |
| 24-36 h | 18 (11.5) | 8 (8.6) |  |
| 36-48 h | 3 (1.9) | 4 (4.3) |  |
| 48 h-1 week | 20 (12.7) | 12 (12.9) |  |
| >1week | 7 (4.5) | 3 (3.2) |  |
| END – no. (%) | 6 (3.8) | 23 (24.7) | <0.001 |
| Acute treatment – no. (%) |  |  |  |
| IV tPA | 4 (2.5) | 8 (8.6) | 0.061^c^ |
| IA Thrombectomy | 4 (2.5) | 6 (6.5) | 0.181^c^ |
| Hypertension – no. (%) | 81 (51.6) | 44 (47.3) | 0.513 |
| Diabetes mellitus – no. (%) | 40 (25.5) | 22 (23.7) | 0.747 |
| Atrial fibrillation – no. (%) | 18 (11.5) | 13 (14.0) | 0.56 |
| Previous history – no. (%) |  |  |  |
| Stroke – no. (%) | 20 (12.7) | 21 (22.6) | 0.042 |
| WBC – 10^9^/L | 7.7 ± 2.6 | 8.3 ± 2.9 | 0.115 |
| Hemoglobin – g/dL | 13.9 ± 1.9 | 13.4 ± 1.9 | 0.046 |
| PT – INR | 1.0 ± 0.2 | 1.0 ± 0.2 | 0.519 |
| Serum glucose – mg/dL | 144.1 ± 58.6 | 141.4 ± 50.5 | 0.715 |
| BUN – mg/dL | 16.4 ± 5.8 | 17.7 ± 7.3 | 0.105 |

### c) External dataset B

|  | **Favorable outcome** | **Unfavorable outcome** |  |
| --- | --- | --- | --- |
| **Variables** | (N = 78) | (N = 32) | P-value |
| Age – year | 66.8 ± 12.4 | 76.3 ± 11.1 | <0.001 |
| BMI – kg/m^2^ | 24.0 ± 3.2 | 24.0 ± 3.6 | 0.955 |
| NIHSS score  at baseline (IQR) | 2 (1-4) | 6 (4-12.3) | <0.001^a^ |
| Onset to admission delay – no. (%) |  |  | 0.77^b^ |
| < 3 h | 29 (37.2) | 9 (28.1) |  |
| 3-6 h | 16 (20.5) | 8 (25.0) |  |
| 6-12 h | 13 (16.7) | 7 (21.9) |  |
| 12-24 h | 7 (9.0) | 6 (18.8) |  |
| 24-36 h | 3 (3.8) | 0 (0.0) |  |
| 36-48 h | 3 (3.8) | 1 (3.1) |  |
| 48 h-1 week | 6 (7.7) | 1 (3.1) |  |
| >1week | 1 (1.3) | 0 (0.0) |  |
| END – no. (%) | 2 (2.6) | 10 (31.3) | <0.001^c^ |
| Acute treatment – no. (%) |  |  |  |
| IV tPA | 9 (11.5) | 4 (12.5) | 1.000^c^ |
| IA Thrombectomy | 8 (10.3) | 6 (18.8) | 0.227^c^ |
| Hypertension – no. (%) | 40 (51.3) | 24 (75.0) | 0.022 |
| Diabetes mellitus – no. (%) | 25 (32.1) | 11 (34.4) | 0.814 |
| Atrial fibrillation – no. (%) | 20 (25.6) | 12 (37.5) | 0.214 |
| Previous history – no. (%) |  |  |  |
| Stroke – no. (%) | 4 (5.1) | 3 (9.4) | 0.413^c^ |
| WBC – 10^9^/L | 7.9 ± 3.1 | 8.4 ± 1.9 | 0.39 |
| Hemoglobin – g/dL | 14.5 ± 1.9 | 13.8 ± 1.5 | 0.048 |
| PT – INR | 1.0 ± 0.1 | 1.1 ± 0.1 | 0.229 |
| Serum glucose – mg/dL | 139.6 ± 60.2 | 148.6 ± 57.3 | 0.476 |
| BUN – mg/dL | 16.3 ± 5.3 | 18.0 ± 6.1 | 0.147 |

BMI, body mass index; mRS, modified Rankin Scale; NIHSS, National Institutes of Health Stroke Scale; TOAST, Trial of Org 10172 in Acute Stroke Treatment^1^; END, early neurologic deterioration; tPA, tissue plasminogen activator; WBC, white blood cell; PT-INR, prothrombin time international normalized ratio; BUN, blood urea nitrogen; HDL, high-density lipoprotein; LDL, low-density lipoprotein

mRS: Scores on the modified Rankin Scale range from 0 to 6, with higher scores indicating greater disability.

NIHSS: Scores on the National Institutes of Health Stroke Scale (NIHSS) range from 0 to 42, with higher scores indicating greater deficits.

^a^ *P* value obtained using Wilcoxon Signed Rank test

^b^ *P* value obtained using Cochran-Armitage trend test

^c^ *P* value obtained using Fisher’s exact test

## Figure S5. ROC curves generated by the XGB algorithm for the internal set, external set A, and external set B


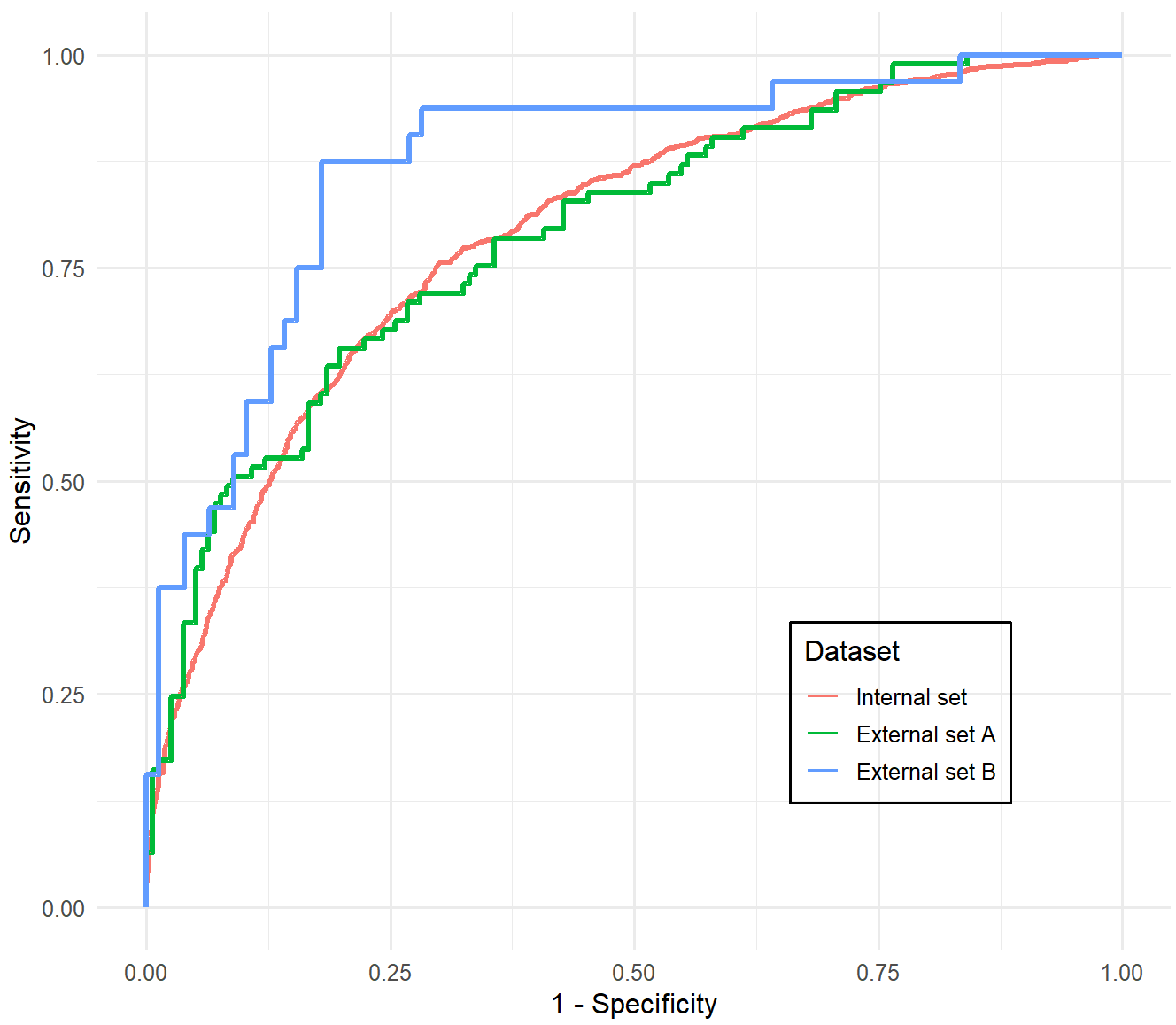


## Figure S6. Calibration plots for the internal set, external set A, and external set B


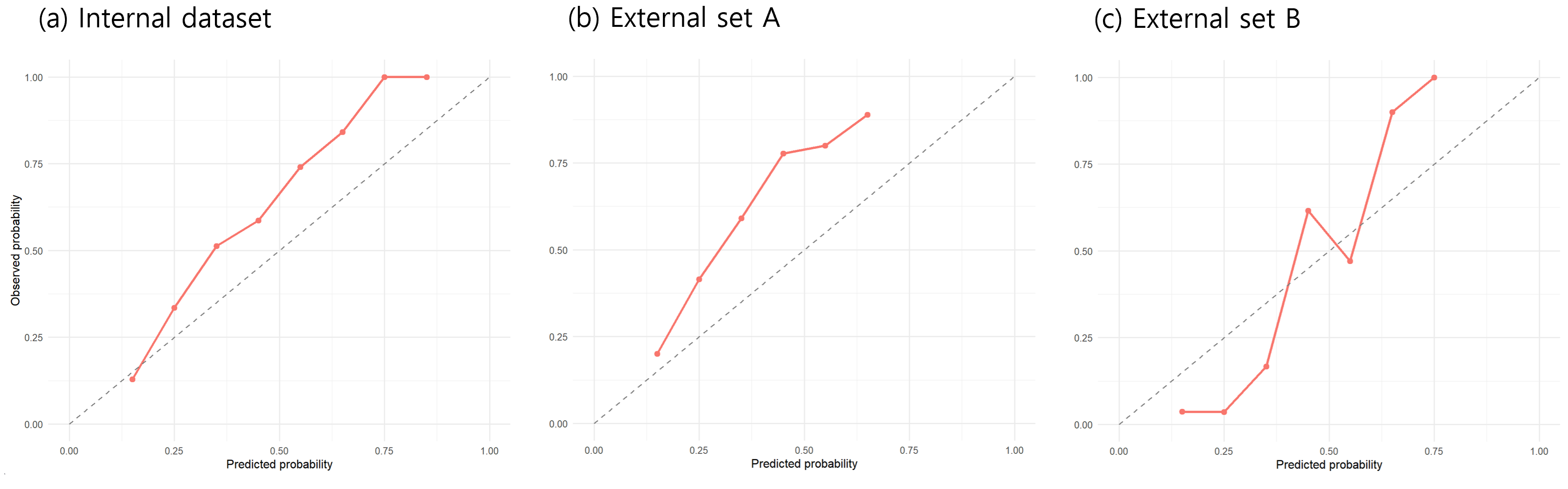


## Figure S7. Interactions between different features and their effects on unfavorable outcome


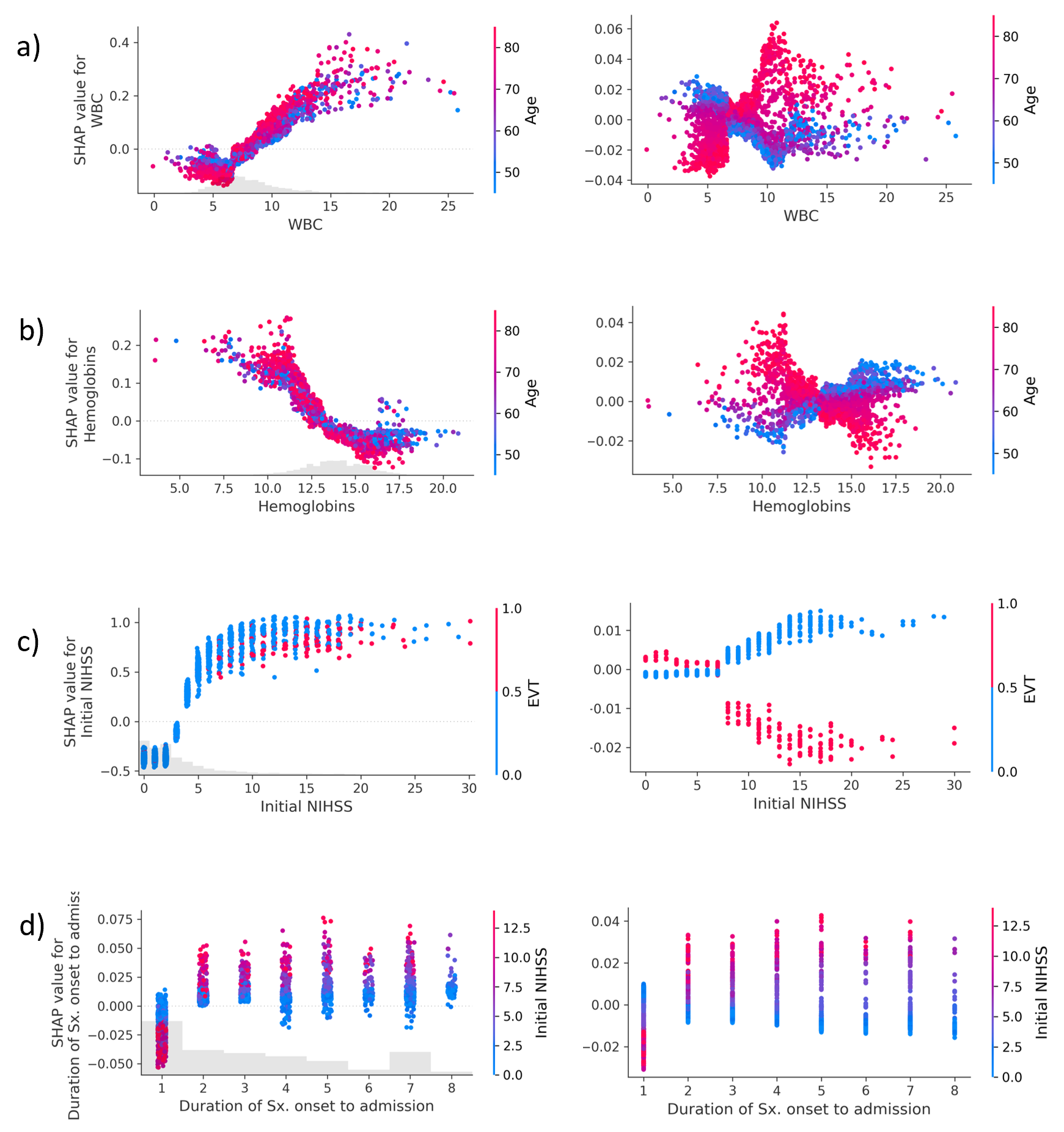


a) The left column of the plot depicts both the main effects and the interaction effects, while the right column isolates and displays only the interaction effects. When Age was approximately over 65 and WBC was less than 7000 /μL, a negative interaction with the unfavorable outcome was observed. However, when WBC exceeded 7000 /μL, a positive interaction was seen. This suggests that when WBC is above 7000 /μL, older patients may be more susceptible to unfavorable outcomes compared to younger patients. b) Contrary to WBC, Hemoglobin has an opposite association with unfavorable outcomes, but the interaction effect with Age is similar. As Hemoglobin decreases, the probability of unfavorable outcome occurrence increases more sharply in older patients compared to younger ones. c) Endovascular Treatment (EVT) was performed when the initial NIHSS was between 6 and 20. The implementation of EVT showed a negative interaction with unfavorable outcome. d) Patients with an initial NIHSS score of approximately 8 or above who arrived within 3 hours showed a negative effect on unfavorable outcomes.

## Figure S8. Local interpretation of individual patient outcome


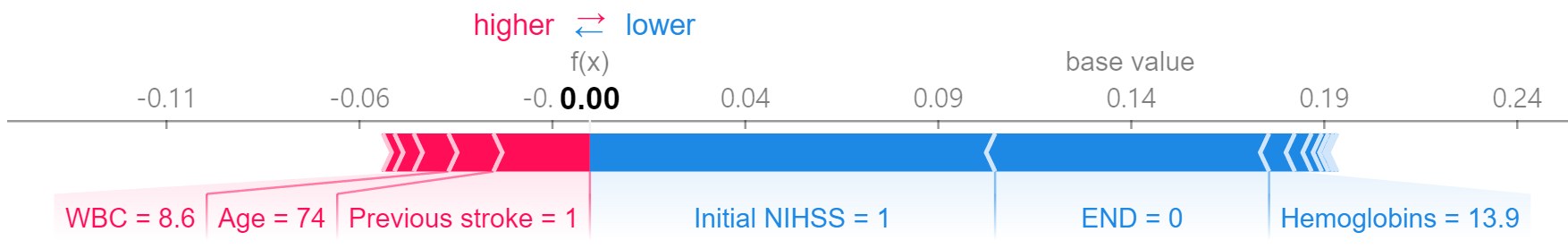


## Figure S9. Forest plot of multivariable logistic regression model


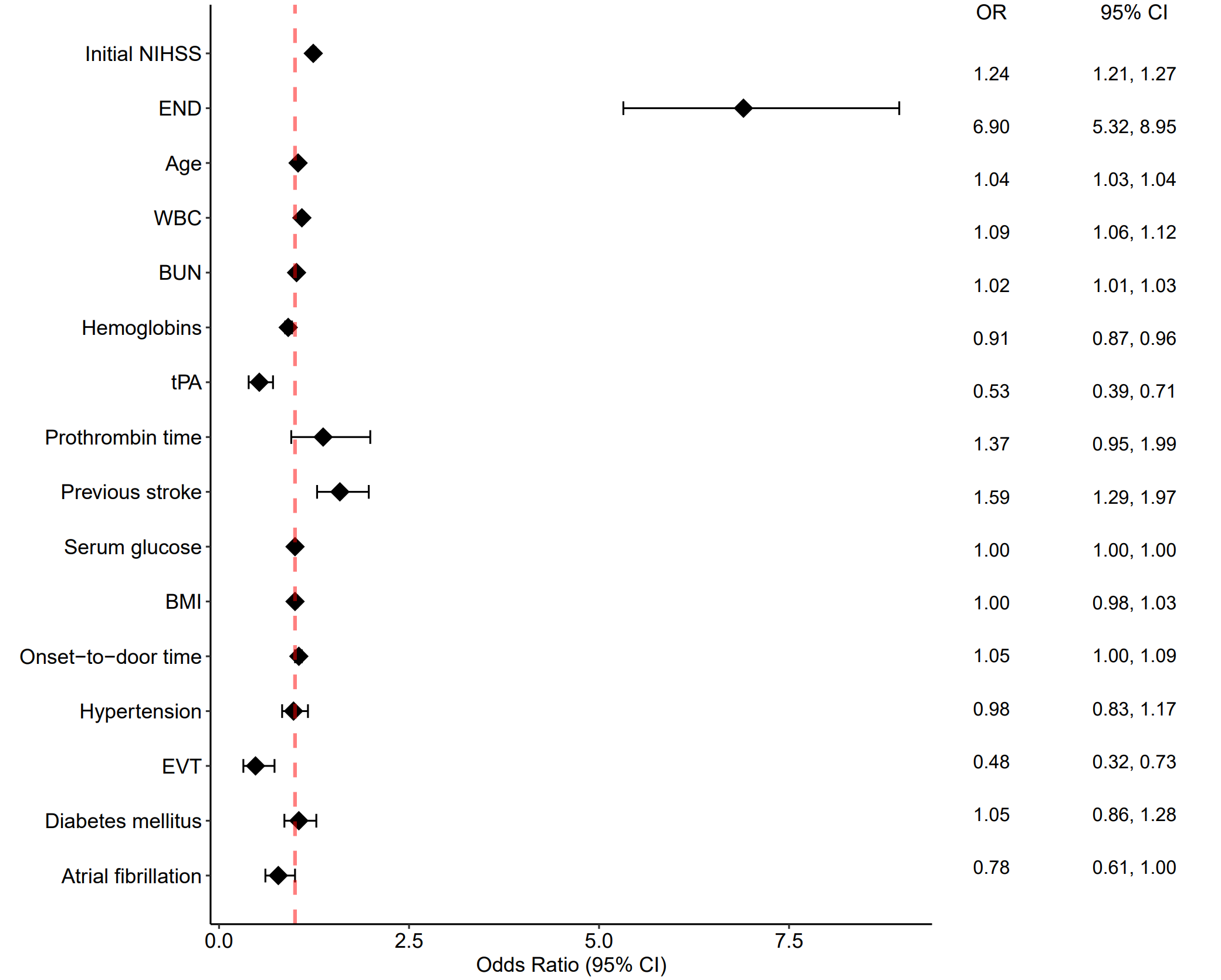


# Reference

1. Adams Jr HP, Bendixen BH, Kappelle LJ, et al. Classification of subtype of acute ischemic stroke. Definitions for use in a multicenter clinical trial. TOAST. Trial of Org 10172 in Acute Stroke Treatment. stroke 1993;24:35-41.

2. Van Buuren S, Groothuis-Oudshoorn K. mice: Multivariate imputation by chained equations in R. Journal of statistical software 2011;45:1-67.

3. Lundberg SM, Erion G, Chen H, et al. From local explanations to global understanding with explainable AI for trees. Nature machine intelligence 2020;2:56-67.
